# Research progress on abdominal epilepsy in children

**DOI:** 10.3389/fnins.2026.1791261

**Published:** 2026-04-02

**Authors:** Wei Meng, Qingyu Xu, Qi Shi, Yi Song, Jiafu Hou

**Affiliations:** 1 Department of Pediatric, Hong Qi Hospital Affiliated to Mudanjiang Medical University, Mudanjiang, Heilongjiang, China; 2 Department of Pediatric Surgery, Hong Qi Hospital Affiliated to Mudanjiang Medical University, Mudanjiang, Heilongjiang, China; 3 Graduate School of Mudanjiang Medical University, Mudanjiang, China; 4 Pharmacy College of Mudanjiang Medical University, Mudanjiang, Heilongjiang, China

**Keywords:** abdominal epilepsy, children, diagnosis, pathogenesis, treatment

## Abstract

Abdominal epilepsy (AE) is a type of focal autonomic epilepsy characterized by recurrent paroxysmal abdominal pain as the primary clinical manifestation. It is relatively more common in children and adolescents and is often accompanied by autonomic nervous system dysfunction and altered consciousness. Due to its atypical clinical presentation and symptom overlap with gastrointestinal disorders, AE is frequently misdiagnosed or overlooked in clinical practice. Advances in neuroimaging, electroencephalography (EEG), and molecular biology have deepened the understanding of AE’s pathogenesis and diversified its diagnostic and therapeutic strategies. This article systematically reviews and critically appraises recent research on AE in children, covering epidemiology, pathogenesis, clinical manifestations, diagnosis, differential diagnosis, treatment, and prognosis. By evaluating the strengths and limitations of the existing evidence (predominantly from case reports, small case series, and a limited number of controlled studies), it aims to highlight reliable conclusions, identify knowledge gaps, and inform future clinical practice and research priorities.

## 1 Introduction

Epilepsy encompasses a broad category of conditions and disorders with diverse etiologies, varied clinical presentations, and significant differences in clinical outcomes (Jing et al., 2022). AE is a special type of focal autonomic epilepsy characterized by recurrent, paroxysmal, and transient abdominal pain as the primary clinical manifestation (Puthanveetil et al., 2021). This condition was first described and reported by Moore in 1944, hence, it is also known as Moore syndrome (Xi et al., 2016). AE is a rare disorder predominantly seen in children. Onset can often be traced to infancy, and the condition is uncommon in adults (Ather et al., 2021). Its clinical presentation is dominated by abdominal pain, which typically lasts from a few minutes to several hours, and may be accompanied by pallor, sweating, vomiting, drowsiness, and symptoms of autonomic nervous system dysfunction (Giuliano et al., 2020). Some patients may experience brief impairment of consciousness or behavioral abnormalities, yet no significant organic pathology is found in the digestive system. This often leads to misdiagnosis as acute abdomen, abdominal migraine, or gastroenteritis, among other conditions. Due to the relatively limited number of case reports and literature, awareness of this disease remains low. In recent years, research in neuroelectrophysiology, neuroimaging, and the gut–brain axis has provided new perspectives for understanding the mechanisms and advancing the precise diagnosis and treatment of AE. This article aims to review relevant domestic and international literature to systematically summarize the current state of research on abdominal epilepsy in children, thereby enhancing clinical recognition and guiding standardized diagnosis and management.

## 2 Methods

This study aimed to systematically review the research progress on abdominal epilepsy in children. A comprehensive literature search was conducted in January 2026 across both English and Chinese databases, such as PubMed, Embase, Web of Science, China National Knowledge Infrastructure (CNKI), and Wanfang Data. The search strategy combined Medical Subject Headings (MeSH) terms and free-text words. English search terms included “abdominal epilepsy,” “visceral epilepsy,” “abdominal pain” AND “epilepsy,” “children,” and “pediatric.” Chinese search terms included “腹型癫痫,” “癫痫,” “腹痛,” and “儿童.” The search timeframe was from the inception of each database to January 2026.

The inclusion criteria were as follows: (1) studies involving children and adolescents (age ≤ 18 years) diagnosed with or discussing AE; (2) study types such as observational studies, case reports/series, clinical trials, and review articles; (3) articles published in English or Chinese. Exclusion criteria were as follows: (1) studies focusing solely on adult populations (age > 18 years) without pediatric data; (2) non-original research (e.g., commentaries and editorials); (3) articles for which the full text was unavailable.

Literature screening and data extraction were performed independently by two reviewers. The selection process involved reviewing titles, abstracts, and full texts. Any discrepancies were resolved through discussion or by consulting a third reviewer. The following information was extracted from the included studies: first author, publication year, study design, sample size, epidemiological characteristics, pathogenesis, clinical manifestations, diagnostic approaches, treatment strategies, and prognostic data. A narrative synthesis was then performed.

## 3 Epidemiological characteristics

AE is a rare disease predominantly seen in childhood, with onset often traceable to infancy, and is uncommon in adults (Ather et al., 2021). Due to its low incidence and susceptibility to misdiagnosis, precise epidemiological data remain limited. Estimates of AE incidence, often derived by extrapolating from case series within broader epilepsy cohorts, are approximately 1–5% (Yong et al., 2025). The disease most frequently occurs in children and adolescents aged 5–15 years, with a peak onset age between 6 and 10 years. No significant racial differences have been observed in AE incidence; however, gender differences have been reported, with a slightly higher incidence in males than females—the male-to-female ratio ranges from approximately 1.15:1 to 1.7:1 (Zhang et al., 2018). Factors such as a family history of epilepsy, febrile seizures, head trauma, or perinatal hypoxia may increase the risk of developing AE. Although cases have been reported in various regions and countries, there is currently no clear evidence indicating regional variations in incidence. It is crucial to note that the current epidemiological understanding is primarily derived from retrospective case series and reports, which are susceptible to publication and referral bias. The absence of large-scale, population-based prospective studies significantly limits the precision of incidence estimates and the ability to definitively assess demographic and geographic risk factors.

## 4 Pathogenesis

### 4.1 Neural network abnormalities

AE is a focal epilepsy whose distinct pathophysiology lies not in a unique epileptogenic mechanism, but in the specific neural networks involved. While the fundamental pathophysiology of epilepsy involves abnormal, excessive, and synchronous neuronal discharges, AE is characterized by discharges that originate from or preferentially engage brain regions responsible for visceral sensation and autonomic function. The epileptogenic focus in AE is commonly located in or involves the temporal lobe (Harshe et al., 2016), particularly limbic structures, such as the hippocampus and amygdala, which are integral to autonomic regulation. Ictal discharges originating from regions such as the insula, frontal lobe (Jie and Xiao, 2025), and postcentral gyrus are also implicated in AE, as these regions are considered higher centers of autonomic function. The insula receives afferent information from visceral organs. When abnormal electrical activity spreads to areas, such as the insula, medial temporal lobe (hippocampal-amygdala complex), orbitofrontal region, and medial prefrontal cortex, episodic autonomic symptoms can manifest (Jia et al., 2024). These abnormal neuronal discharges can influence gastrointestinal function via direct projections to the dorsal motor nucleus of the vagus nerve, leading to gastrointestinal symptoms. The hypothalamus may also contribute to gastrointestinal symptoms by activating sympathetic pathways from the amygdala to the gut (Mpondo et al., 2016). Intracranial electrical stimulation studies have found that the medial prefrontal cortical expansion area has efferent pathways to diencephalic and brainstem autonomic nuclei (such as the nucleus of the solitary tract and the ambiguous nucleus); stimulating this area can induce changes in blood pressure, heart rate, and gastrointestinal motility (Lei et al., 2014). During episodes, levels of inflammatory cytokines (e.g., IL-1β and IL-6) in the cerebrospinal fluid (CSF) are significantly higher than in healthy controls (*p* < 0.01) (Qiu et al., 2017) and show a positive correlation with the frequency of interictal epileptiform discharges on EEG, suggesting that neuroinflammation may be involved in modulating seizure thresholds (Morrow et al., 2025). However, the current evidence cannot definitively establish whether the elevated cytokine levels are a primary cause triggering the abdominal pain episodes or a secondary effect resulting from the neuronal hyperexcitability and associated neural inflammation during the ictal event. Abnormal neuronal discharges originating in the brainstem or limbic system can propagate to higher cortical centers, affecting gastrointestinal regulatory functions and triggering symptoms like abdominal pain (Zhi et al., 2022). The neural networks involved in AE are complex, and the specific pathways of abnormal discharge propagation require further research.

Functional neuroimaging techniques, particularly Positron Emission Tomography (PET) (Dehno et al., 2025) and Single-Photon Emission Computed Tomography (SPECT) (Chen et al., 2025; Azad et al., 2026), have been utilized in a limited number of cases to localize the epileptogenic focus. Ictal SPECT, which measures cerebral blood flow, may show hyperperfusion in regions such as the temporal lobe, insula, or frontal cortex during an abdominal seizure episode. Interictal fluorodeoxyglucose-PET(FDG-PET), which assesses glucose metabolism, might reveal hypometabolism in similar regions, supporting the presence of a functional abnormality in neural networks governing visceral sensation and autonomic function (Li et al., 2026). However, the evidence is sparse, derived from isolated case reports, and the sensitivity and specificity of these modalities, specifically for AE, have not been established through systematic studies.

### 4.2 Neurotransmitter imbalance

Imbalance of neurotransmitters is a key mechanism in the occurrence of AE. Studies have found that neurotransmitters such as serotonin (5-HT), norepinephrine (NE), *γ*-aminobutyric acid (GABA), and glutamate (Glu) are involved in the regulation of abdominal pain and epileptic seizures (Qian et al., 2019). GABA is an inhibitory neurotransmitter in the central nervous system, and its reduced levels can lead to neuronal hyperexcitability, which is associated with epileptic seizures (Zhuo and Qin, 2025; Ying and Yue, 2023). Glutamate (Glu) is a major excitatory neurotransmitter, and its excessive release can contribute to neuronal hyperexcitability and ictogenesis (Hao et al., 2023). In AE patients, the levels of inhibitory neurotransmitters, such as GABA and 5-HT, in the brain are often reduced, while the levels of excitatory neurotransmitters, such as NE and Glu are elevated. This imbalance in neurotransmitter homeostasis leads to abnormal neuronal discharges, thereby triggering episodes of abdominal pain. Additionally, the gut microbiota can influence neurotransmitter levels in the brain by directly secreting neurotransmitters or their metabolites, or by stimulating gastrointestinal cells to produce neurotransmitters (Fei et al., 2025). For example, certain species of *Lactobacillus* and *Bifidobacteriumcan* produce GABA, while some strains of *Escherichia coli* and *Bacillus* are involved in the synthesis of NE and dopamine. This suggests that neurotransmitter dysregulation in AE may be associated with the interaction between the central nervous system and the gut.

### 4.3 Genetic factors

Genetic factors may contribute to the occurrence of AE, but the specific genetic patterns and related gene loci remain unclear. Currently, there are no reports identifying definitive pathogenic genes for AE. It is hypothesized that AE may be a polygenic disorder, where interactions among multiple genes could increase disease susceptibility. Additionally, some genetic disorders associated with epilepsy may present with abdominal clinical manifestations, potentially linked to mutations in genes involved in neural development or neurotransmitter synthesis (Zhao et al., 2025). For example, mutations in ion channel-related genes may lead to abnormal neuronal excitability, which might play a role in the development of AE. However, the role of genetic factors in AE requires further in-depth genetic studies for confirmation.

### 4.4 Environmental factors

Environmental factors may be significant triggers for AE. Infection is one of the common environmental factors (Xue et al., 2025); conditions such as viral encephalitis and bacterial meningitis can cause brain inflammation and neuronal damage, potentially increasing the risk of AE onset (Ye et al., 2025). Metabolic disturbances, such as hypoglycemia and hypocalcemia, can interfere with normal neuronal function, leading to abnormal discharges and triggering AE episodes. A single-center study on benign convulsions associated with mild gastroenteritis found that serum calcium levels were significantly lower in patients with multiple episodes compared to those with a single episode, suggesting that hypocalcemia may be associated with both the occurrence and frequency of seizures (Xiao Y. Q. et al., 2025; Luo et al., 2025). Additionally, head trauma can cause structural abnormalities or functional impairments in the brain, thereby increasing the risk of developing epilepsy, such as AE (Hong et al., 2023). Regarding this specific question: If recurrent paroxysmal abdominal pain emerges as the primary or soleictal manifestation following head trauma and meets other diagnostic considerations for AE (e.g., EEG correlates and response to AEDs), the condition could be clinically described as post-traumatic epilepsy with focal autonomic (abdominal) seizures. It is more accurate to classify such cases based on etiology (post-traumatic) within the broader epilepsy classification framework, rather than labeling it broadly as “abdominal epilepsy,” which is typically considered a seizure type or a presentation that can occur within various etiologies. Certain medications, such as antibiotics and antidepressants, may have pro-convulsant effects and could induce AE in susceptible individuals. Chronic stress is also a potential environmental factor; it can affect the gut–brain axis, leading to disruptions in gut microbiota, which may subsequently increase susceptibility to epilepsy.

### 4.5 Role of the gut–brain axis

In recent years, the role of the gut–brain axis (GBA) in the development and progression of neurological disorders has garnered increasing attention. The GBA is a bidirectional communication system between the gastrointestinal tract and the brain, regulating gut homeostasis and central nervous system function through neural, immune, endocrine, and metabolic pathways (Lu et al., 2023). Research suggests that the GBA may be involved in the pathogenesis of AE. On one hand, gut microbiota dysbiosis can disrupt the intestinal barrier, allowing pathogens to translocate into the portal vein and systemic circulation, triggering neuroinflammation and subsequently inducing abnormal neuronal discharges (Xi et al., 2023). On the other hand, gut microbiota can directly secrete neurotransmitters or produce metabolites, such as short-chain fatty acids, modulating central neurotransmitter levels and influencing neuronal excitability (Yuan and Jie, 2022), thereby contributing to the occurrence of AE. For example, colonization of the gastrointestinal tract with *Akkermansia* and *Parabacteroides* species in antibiotic-treated mice reduced levels of excitatory amino acids, such as glutamate in the serum and gut lumen, while increasing GABA expression in the hippocampus, exerting an antiepileptic effect (Xiao L. D. et al., 2025). Additionally, the vagus nerve serves as a key pathway for bidirectional communication within the GBA; microbial metabolites can activate the vagus nerve, transmitting gut-derived signals to the brain and potentially influencing seizure occurrence.

**Figure 1:**
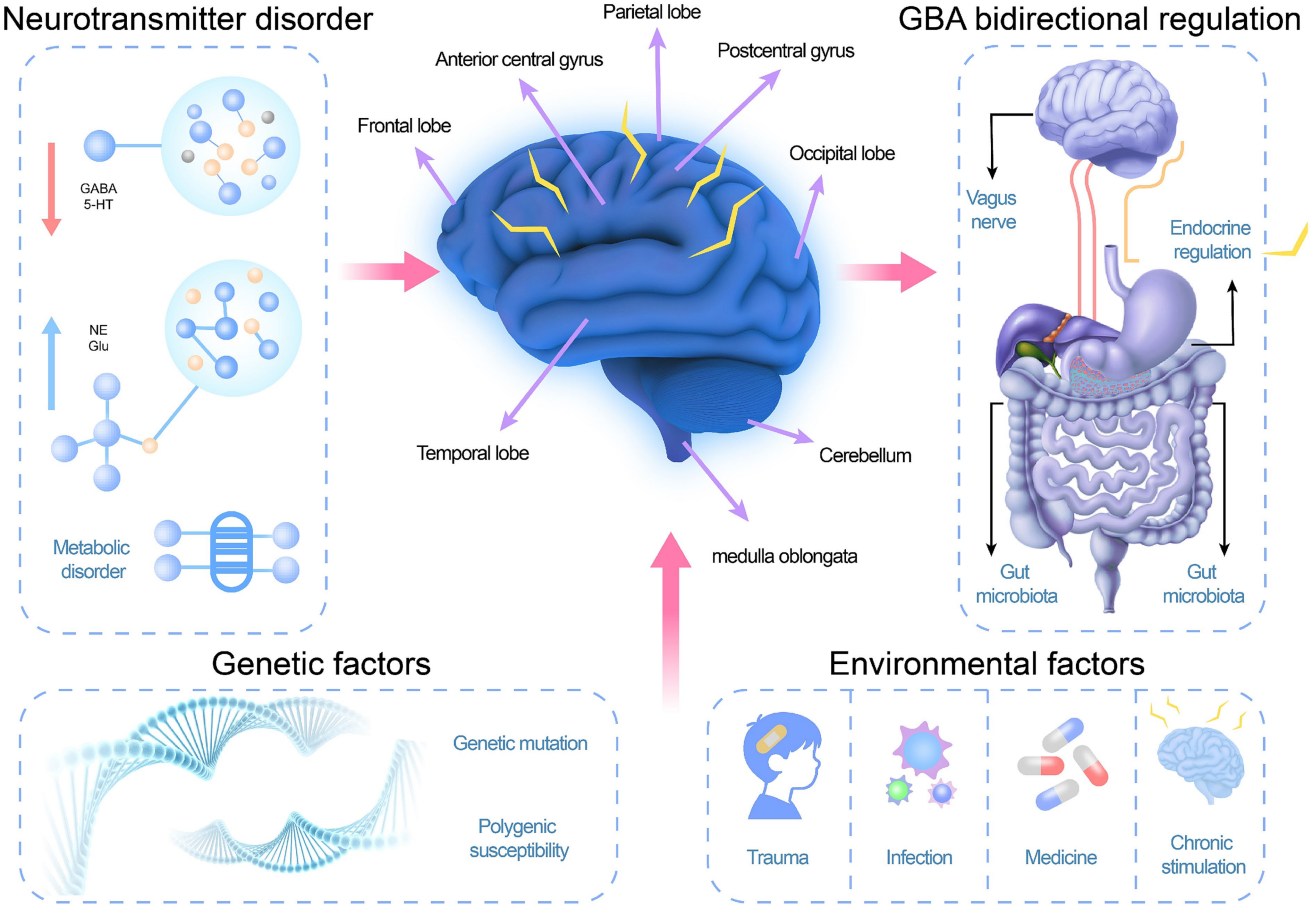
Schematic diagram of the pathogenesis of abdominal epilepsy in children (image created with Adobe Illustrator).

However, regarding the extent to which this mechanism is established in patients, it is crucial to note that the evidence remains preliminary. There is currently no established figure or percentage of AE patients in whom a GBA dysfunction has been definitively proven as the primary cause. The findings implicating the GBA in AE are predominantly from preclinical animal models and correlative human studies (e.g., observing microbiota differences in epilepsy cohorts). The causal relationship between specific gut microbiota alterations and the onset of AE in humans remains to be established through prospective, interventional studies. A schematic diagram illustrating these mechanisms is shown in Figure 1.

## 5 Clinical manifestations

The typical presentation of AE is characterized by sudden, recurrent, and paroxysmal abdominal pain (Rakesh et al., 2014). The pain is usually localized around the umbilicus or in the upper abdomen, and may occasionally radiate to the lower abdomen or flanks. It is often described as colicky or cramping, with an intensity ranging from moderate to severe; visual analog scale (VAS) scores can reach up to 7. The episodes last from several minutes to several hours, begin and end abruptly, and occur with a frequency varying from several times a day to several times a month. Autonomic nervous system symptoms are common during attacks, such as pallor, sweating, cold extremities, mydriasis (pupil dilation), nausea, vomiting, and loss of appetite (Giuliano et al., 2020). Nausea and vomiting are particularly frequent, and patients with multiple episodes exhibit a significantly higher incidence of vomiting compared to those with single episodes. Ictal changes in blood pressure and heart rate, such as hypertension and tachycardia, may also occur. These vital sign alterations are part of the autonomic dysfunction and can support the diagnosis, but they are not specific to abdominal epilepsy. Some patients may experience brief disturbances of consciousness, such as drowsiness or confusion, while a minority may present with motor symptoms like limb twitching or nystagmus (Anvesh et al., 2020). Consciousness is typically clear or only mildly impaired during the attack. Most patients experience postictal fatigue, drowsiness, or deep sleep, awakening afterward feeling well. Neurological examinations usually reveal no significant positive findings. Based on the primary clinical features, AE can be broadly categorized into two main subtypes: one characterized primarily by cyclic vomiting and the other by cyclic abdominal pain. In the cyclic vomiting subtype, typical episodes involve vomiting that lasts 20–40 min and may occur multiple times daily or every few days. The cyclic abdominal pain subtype typically features abdominal pain lasting several minutes to tens of minutes; this may remain as isolated pain or potentially evolve into a generalized seizure.

## 6 Auxiliary examinations

### 6.1 Electroencephalogram (EEG) examination

EEG is a crucial tool for diagnosing AE (Ting, 2025a). Abnormal EEG waveforms in children with AE primarily include isolated spikes, sharp-and-slow wave complexes, paroxysmal fast or slow waves, diffuse fast or slow waves, as well as positive spikes (Jagtap et al., 2018). EEG findings often show focal abnormalities in the temporal lobe. Capturing a synchronous occurrence of abdominal pain and epileptiform discharges on long-term video-EEG (vEEG) is key for a definitive diagnosis and represents a typical feature of this condition (Kshirsagar et al., 2012).

### 6.2 Imaging examinations

Head magnetic resonance imaging (MRI) helps rule out structural lesions, such as brain tumors, cerebrovascular malformations, and abnormal brain development (Vikin et al., 2025). For children with AE of unknown etiology, a head MRI is recommended to identify potential causes (Song Y. et al., 2025). In cases with a history of head trauma, computed tomography (CT) scans of the head may be necessary to further investigate related pathologies (Kui, 2025).

In cases where conventional MRI is non-lesional or the epileptogenic focus remains elusive, functional neuroimaging modalities like PET and ictal/interictal SPECT can provide adjunctive localizing information. These techniques assess cerebral metabolism (FDG-PET) or perfusion (SPECT) and may identify functional abnormalities in brain regions implicated in AE, such as the temporal lobe, insula, or cingulate cortex (Chen et al., 2025; Azad et al., 2026). Ictal SPECT, performed during an abdominal episode, aims to capture the area of hyperperfusion associated with the seizure onset zone. Interictal FDG-PET may show focal hypometabolism (Li et al., 2026). However, its application in AE is not routine and is primarily documented in case reports or small series within the broader context of focal epilepsies presenting with autonomic or visceral symptoms. The availability, need for tracer injection close to the often brief and unpredictable abdominal episodes, and the lack of standardized interpretation criteria for AE limit their widespread use in clinical practice.

### 6.3 Other examinations

Investigations such as abdominal ultrasound, gastroscopy, colonoscopy, and gastrointestinal contrast studies are required to exclude organic diseases of the digestive system. Blood tests, such as complete blood count, liver and kidney function, and electrolytes, are useful for assessing the child’s overall health status and monitoring medication safety. See Table 1 for a summary.

**Table 1:** Auxiliary examination methods for abdominal epilepsy and their clinical value.

Inspection category	Specific method	Main purpose	Typical positive findings/indicative significance	Reference
Electrophysiological examination	EEG	Initial screening, capturing interictal discharges.	Epileptic discharges, such as spike waves, sharp waves, and spike-and-wave complexes, may be observed, though the yield of routine interictal EEG is low.	Jagtap et al. (2018)
	Long-term vEEG	Critical for definitive diagnosis, allowing for synchronous recording of symptoms and EEG.	Epileptiform discharges during the seizure phase, with clear synchronization between symptoms and discharges.	Tatum et al. (2020), Tong and Lin (2025)
Neuroimaging	Head MRI	To exclude structural etiologies and identify potential epileptogenic foci.	Brain tumors, cerebral vascular malformations, hippocampal sclerosis, cortical dysplasia, etc.	Fares et al. (2025), Fan et al. (2025)
	Head CT	Emergency situation, excluding acute bleeding, calcification, etc.	Acute intracranial lesions (such as hemorrhage, extensive infarction).	Takala et al. (2025), Xian et al. (2023)
Digestive system examination	Abdominal ultrasound	Excluding lesions of abdominal organs.	Appendicitis, intussusception, intestinal obstruction, etc.	Hosale et al. (2023), Sangüesa-Nebot and Llorens-Salvador (2021)
	Gastroscopy	Excluding gastrointestinal mucosal lesions.	Gastritis, peptic ulcer, inflammatory bowel disease, etc.	Ravikumar et al. (2025), Zhang et al. (2025)

## 7 Diagnosis and differential diagnosis

### 7.1 Diagnostic criteria

A major challenge in AE research is the absence of universally accepted, validated diagnostic criteria. Current proposed criteria, often synthesized from small case series and expert opinion, include: ① Recurrent paroxysmal abdominal pain, characterized by sudden onset and transient episodes, in the absence of significant organic gastrointestinal pathology (Arisa et al., 2016). ② Epileptiform abnormalities on EEG—such as spikes, sharp waves, or spike-and-slow-wave complexes—during ictal or interictal periods serve as key diagnostic evidence (Öztürk et al., 2019). However, as some patients may show normal interictal EEG, long-term VEEG monitoring is recommended to capture abnormalities during episodes, significantly improving diagnostic accuracy. ③ Marked relief or resolution of abdominal pain following antiepileptic drugs (AEDs) therapy (Harika et al., 2016). ④ Exclusion of other causes of paroxysmal abdominal pain through appropriate investigations. Neuroimaging (e.g., brain MRI or CT) may be used to rule out intracranial lesions such as tumors, vascular malformations, or injuries. These criteria emphasize the integration of clinical presentation, electrophysiological findings, treatment response, and exclusion of alternative diagnoses (Kui, 2025). It is important to note that the evidence supporting this diagnostic framework is of low quality. Reliance on AED response introduces diagnostic circularity. Furthermore, the sensitivity of routine EEG is limited, and the specificity of EEG abnormalities in the context of abdominal pain is not well-established. The diagnosis often rests on a combination of suggestive features rather than a pathognomonic test, underscoring the need for more rigorous diagnostic validation studies.

### 7.2 Differential diagnosis

Since abdominal epilepsy primarily manifests as abdominal pain, it must be differentiated from various other conditions that present with abdominal pain. The key to differentiation lies in a detailed analysis of the characteristics of the abdominal pain, associated symptoms, relevant examination findings, and the response to specific treatments, as outlined in Table 2.

**Table 2:** Key differential diagnoses of AE in children.

Disease	Clinical characteristics and differential diagnosis points	Key auxiliary examination	Response to AED treatment	Reference
Acute appendicitis	Paroxysmal metastatic right lower abdominal pain, accompanied by tenderness and rebound tenderness at McBurney’s point, and severe muscle tension, sometimes accompanied by fever and elevated white blood cell count.	Abdominal ultrasound/CT revealed abnormal thickening of the appendix and peritoneal effusion. The electroencephalogram was normal.	Ineffective	Almalki et al. (2025)
Intussusception	It is more common in infants and young children, who may experience paroxysmal crying, vomiting, and jelly-like stools due to pain. A mass can be palpated in the abdomen.	Abdominal ultrasound revealed the “target ring sign,” while the electroencephalogram showed no epileptiform discharges.	Ineffective	Suh et al. (2025)
Intestinal obstruction	Abdominal pain, abdominal distension, vomiting, and cessation of bowel movements and gas passing.	An X-ray of the abdomen reveals a gas-fluid level, while the EEG shows no abnormalities.	Ineffective	Hua et al. (2025), Hencke et al. (2024)
Acute gastroenteritis	Abdominal pain often accompanies diarrhea, nausea, and vomiting, and is often related to a history of unclean eating. It may also be accompanied by fever. Symptoms improve as the infection is controlled.	The EEG showed no epileptiform discharges.	Ineffective	Palorath et al. (2025), Roskind et al. (2025)
Cyclic vomiting syndrome	Recurrent and stereotyped episodes of vomiting, with normal intervals between episodes and no obvious abdominal pain.	The EEG showed no epileptiform discharges.	Ineffective	Hernandez and Gardner (2025), Aziz (2025)
Ascariasis	Paroxysmal colic around the umbilicus, which can resolve spontaneously. A cord-like mass can be palpated in the abdomen. There is a history of worm expulsion or passage, and deworming treatment is effective.	Fecal examination revealed the presence of parasite eggs; electroencephalogram showed no abnormal discharge.	Ineffective	Sorokman and Moldovan (2019)
Abdominal migraine	Paroxysmal abdominal pain, lasting for a long time (from several hours to several days), often accompanied by a family history of migraine, and may present with symptoms such as photophobia and sound aversion.	EEG is normal.	Usually ineffective, but anti-migraine medication is effective	Sahu et al. (2025)
Other types of epilepsy (e.g., temporal lobe epilepsy)	Abdominal discomfort or abnormal sensations may serve as seizure precursors, followed by typical epileptic symptoms such as impaired consciousness, automatism, and convulsions.	VEEG can record epileptiform discharges originating from areas such as the temporal lobe.	Effective	Hua et al. (2024)
Electrolyte imbalance	It can cause abdominal pain and convulsions, but is usually accompanied by other symptoms of corresponding metabolic disorders. Symptoms can be alleviated after correcting the metabolic disorders.	Blood tests can reveal clear abnormalities in metabolic indicators; the electroencephalogram shows no specific discharge.	Ineffective	Zieg et al. (2024)
Porphyria	Acute episodic abdominal pain, neurological symptoms, and psychiatric symptoms, with urine turning dark red after exposure to sunlight.	The diagnosis can be confirmed by measuring urine bilirubin; the electroencephalogram is normal.	Ineffective	Jin et al. (2024), Song J. Z. et al. (2025)
Abdominal aortic dissection	Sudden, intense, tearing-like chest, back, or abdominal pain, accompanied by vascular signs such as abnormal blood pressure and shock. It is more common in adults with a history of hypertension.	Vascular imaging (CTA) can confirm the diagnosis.	Ineffective	Li et al. (2025)
Visceral form of herpes zoster	Before the rash appears, there may be unilateral severe pain, which is distributed along the course of the nerve.	EEG showed no epileptiform discharges.	Ineffective	Timotijevic et al. (2025)

## 8 Treatment

### 8.1 Pharmacotherapy

The management of AE is primarily extrapolated from general epilepsy principles and observational data in AE, as no randomized controlled trials (RCTs) have been conducted specifically for AE. The primary goals are to control seizures and alleviate abdominal pain. AEDs are considered the first-line treatment based on extrapolation from their efficacy in other focal epilepsies, supported by clinical experience and numerous case reports showing positive responses in AE. Drug therapy should follow an individualized approach, starting with a low dose and gradually increasing to an effective level. Regular monitoring of drug plasma concentrations, as well as liver and kidney function and complete blood count, is necessary. Most pediatric patients respond well to pharmacotherapy. If monotherapy proves ineffective, combination therapy may be considered. Medication typically needs to be continued for several years. Only after symptoms are completely controlled can a gradual reduction and discontinuation of the drugs be attempted under a doctor’s guidance to avoid triggering status epilepticus from abrupt withdrawal. Commonly used medications include Valproate, Carbamazepine, Phenytoin, Phenobarbital (Wei et al., 2025), Levetiracetam, and Oxcarbazepine (Yu L. et al., 2025). Among them, Valproate, a broad-spectrum antiepileptic drug, is one of the preferred choices for treating abdominal epilepsy. It works by increasing GABA levels in the brain to suppress abnormal neuronal discharges and is effective against various seizure types. Levetiracetam offers advantages, such as good tolerability and fewer drug interactions. It is suitable for children and adolescents, effective for both partial and generalized seizures, and can be used as a first-line agent or in combination with other drugs (Xin, 2026). Topiramate may be combined in cases where anxiety or migraine overlaps. Oxcarbazepine and Carbamazepine are effective for partial seizures and can also be used for abdominal epilepsy. However, monitoring drug plasma concentrations and watching for adverse effects, such as rash and liver damage are crucial. A detailed comparison is provided in Table 3. It is imperative to recognize that the recommendations in this table are primarily based on open-label studies, retrospective case series, and extrapolation from their use in other focal epilepsy types. The comparative efficacy and safety profile of these AEDs, specifically for AE, are not established by high-level evidence.

**Table 3:** Comparison of commonly used antiepileptic drugs for abdominal epilepsy in children.

Drug name	Mechanism of action/type	Therapeutic characteristics	Common adverse reactions	Precautions	Reference
Sodium valproate	Broad-spectrum, increases GABA levels.	First-line choice, effective for multiple seizure types.	Weight gain, tremor, hair loss, hepatotoxicity, teratogenicity.	Blood drug concentration, liver function, and blood routine examination need to be monitored; use with caution in women of childbearing age.	Trocha et al. (2025)
Levetiracetam	Broad-spectrum, with a unique mechanism of action.	Good tolerance and few drug interactions.	Irritability, aggressive behavior, lethargy, dizziness.	Close observation is necessary for changes in children’s behavior and emotions.	Jia et al. (2025)
Oxcarbazepine	Sodium channel blocker.	Effective for partial seizures.	Drowsiness, dizziness, hyponatremia, and rash.	The blood sodium level needs to be monitored, and attention should be paid to cross-allergic reactions.	Qin et al. (2025)
Carbamazepine	Sodium channel blocker.	Effective for partial seizures.	Dizziness, rash, leukopenia, hyponatremia, and hepatic enzyme induction.	Blood drug concentration, blood routine examination, and serum sodium levels need to be monitored; it is a strong inducer of liver enzymes.	Da et al. (2023)
Phenytoin sodium	Sodium channel blocker.	Effective for tonic–clonic seizures and partial seizures.	Ataxia, nystagmus, gingival hyperplasia, hirsutism, bone marrow suppression, and hepatic enzyme induction.	Blood drug concentration needs to be monitored; nonlinear pharmacokinetics, prone to toxicity; attention should be paid to the impact on bone metabolism during long-term use.	Can et al. (2023)
Phenobarbital	Enhancing GABA can inhibit.	Broad-spectrum antiepileptic effect.	Sedation, somnolence, cognitive and behavioral effects (such as hyperactivity, irritability), and induction of liver enzymes.	Blood drug concentration needs to be monitored; children are prone to behavioral side effects; long-term use affects cognition.	Bo et al. (2025), Fernandes et al. (2025)
Topiramate	Multiple mechanisms (sodium channel, GABA, etc.).	It may be beneficial for children with concomitant migraine or anxiety.	Cognitive slowing, weight loss, kidney stones, sensory abnormalities.	Pay attention to the impact on cognitive function and encourage drinking more water.	Zhong and Le (2025)

### 8.2 Ketogenic diet therapy

For patients with drug-resistant AE—specifically, those whose seizures remain uncontrolled after treatment with two or more anti-epileptic drugs—ketogenic diet therapy may be considered (Hua and Yi, 2025; Wen et al., 2025). The evidence for the ketogenic diet in AE specifically is sparse, consisting of a few case reports and small series. While the ketogenic diet has proven efficacy in other drug-resistant epilepsy syndromes through more robust studies, its application in AE remains supported by very low-quality evidence, and its use should be considered experimental within this specific indication. The ketogenic diet is a dietary regimen characterized by high fat, very low carbohydrate, and adequate protein intake. It works by inducing a metabolic state in which the body produces ketone bodies, which can suppress abnormal neuronal discharges and reduce seizure frequency. In a study by Xin et al. (2025), experiments on mouse models demonstrated that standardized ketogenic diet intervention effectively maintained therapeutic ketosis levels and significantly suppressed abnormal electrical activity in epileptic model animals. Research further indicates that ketogenic diet therapy is effective for some patients with drug-resistant epilepsy and may, to a certain extent, improve cognitive function (Na et al., 2025). However, the ketogenic diet involves strict dietary restrictions and can lead to adverse effects such as constipation, vomiting, and growth retardation. Therefore, it must be implemented under the guidance of professional physicians and dietitians, with regular monitoring of blood glucose, lipid profiles, and ketone body levels (Huan et al., 2025).

### 8.3 Surgical treatment

For patients with drug-resistant AE in whom the epileptogenic focus has been clearly identified through neuroimaging and electrophysiological examinations, surgical intervention may be considered (Wei and Xun, 2025). It must be emphasized that surgery is not a standard treatment for abdominal epilepsy. Existing literature indicates that only a small fraction of drug-resistant patients ultimately require surgical intervention. For instance, one study on surgery for abdominal epilepsy mentioned that surgical patients constituted about 3.8% of all abdominal epilepsy patients evaluated in their case series (Yong et al., 2025). This suggests that the vast majority of patients can be effectively managed with medication. Common surgical approaches include vagus nerve stimulation (VNS), chronic cerebellar stimulation, and stereotactic brain ablation. Vagus nerve stimulation is a minimally invasive procedure that reduces seizure frequency by stimulating the vagus nerve; it is suitable for patients who are not candidates for resection of the epileptogenic focus or in cases where the focus is not well-defined (Kraimer et al., 2019). Stereotactic brain ablation can precisely destroy the epileptogenic zone but requires accurate localization of the focus and carries surgical risks such as intracranial hemorrhage and infection (Bhole et al., 2025; Jian et al., 2021). The benefit of surgery lies in the fact that for carefully selected cases, targeted surgical intervention (such as focal resection or neuromodulation) based on precise localization of the “temporal-limbic-insular” epileptogenic network (through integrated assessment using VEEG, MRI, PET/SPECT, stereotactic electroencephalography, etc.) can significantly reduce or even completely control epileptic seizures, thereby improving patient prognosis and quality of life. Therefore, surgical treatment must be rigorously evaluated by a specialized epilepsy team to develop a tailored surgical plan based on the patient’s specific condition. By integrating data from multiple diagnostic techniques such as VEEG, MRI, functional imaging (e.g., PET, or ictal SPECT), and stereo-electroencephalography (SEEG), a core epileptogenic network involving the “temporal-limbic-insular” regions has been preliminarily established for AE. These functional imaging tools can contribute to focus localization in the pre-surgical evaluation of drug-resistant cases. This approach improves diagnostic accuracy and enables the formulation of individualized treatment plans guided by the characteristics of this network. Surgical interventions based on this strategy have significantly enhanced both the diagnosis and treatment outcomes for abdominal epilepsy (Yong et al., 2025).

### 8.4 Traditional Chinese medicine treatment

Traditional Chinese Medicine (TCM) believes that abdominal epilepsy falls under the category of “abdominal epilepsy syndrome”. Some TCM approaches are explored as adjunctive therapies, with the proposed benefits of mitigating the side effects of AEDs. In clinical practice, traditional Chinese medicine, acupuncture, and moxibustion are often used as adjuvant measures (Jin et al., 2020), combined with antiepileptic drugs, which may enhance efficacy, reduce drug dosage, and mitigate side effects. It can not only fundamentally treat the disease but can simultaneously regulate the functions of the body’s organs and enhance the body’s disease resistance, which are reported to have minimal adverse reactions. It effectively alleviates the severity of seizures, reduces the frequency of seizures, and mitigates the adverse reactions of Western medicine treatment (Zhong et al., 2025). Ying et al. (2020) experimentally confirmed that the decoction of Shaoyao Gancao Tang can increase the content of ATPase and SOD in the brain tissue of epileptic mice, reduce the content of MDA, and exert an inhibitory effect on seizures. Ya and Feng (2024) fully leveraged the superiority of the integrated traditional Chinese and Western medicine approach, combining Liujunzi Decoction with valproate sodium, compensating for each other’s deficiencies and enhancing the synergistic effect on epilepsy treatment. The evidence specifically for AE is extremely limited, typically based on small, uncontrolled case series or reports. While these approaches may offer a complementary management strategy, claims regarding their efficacy in AE are not supported by rigorous clinical trial data. The cited experimental studies (Ying et al., 2020; Ya and Feng, 2024) are preclinical (in animal models), and their results cannot be directly translated to clinical efficacy in human AE without further validation.

### 8.5 Other adjuvant therapies

**Figure 2:**
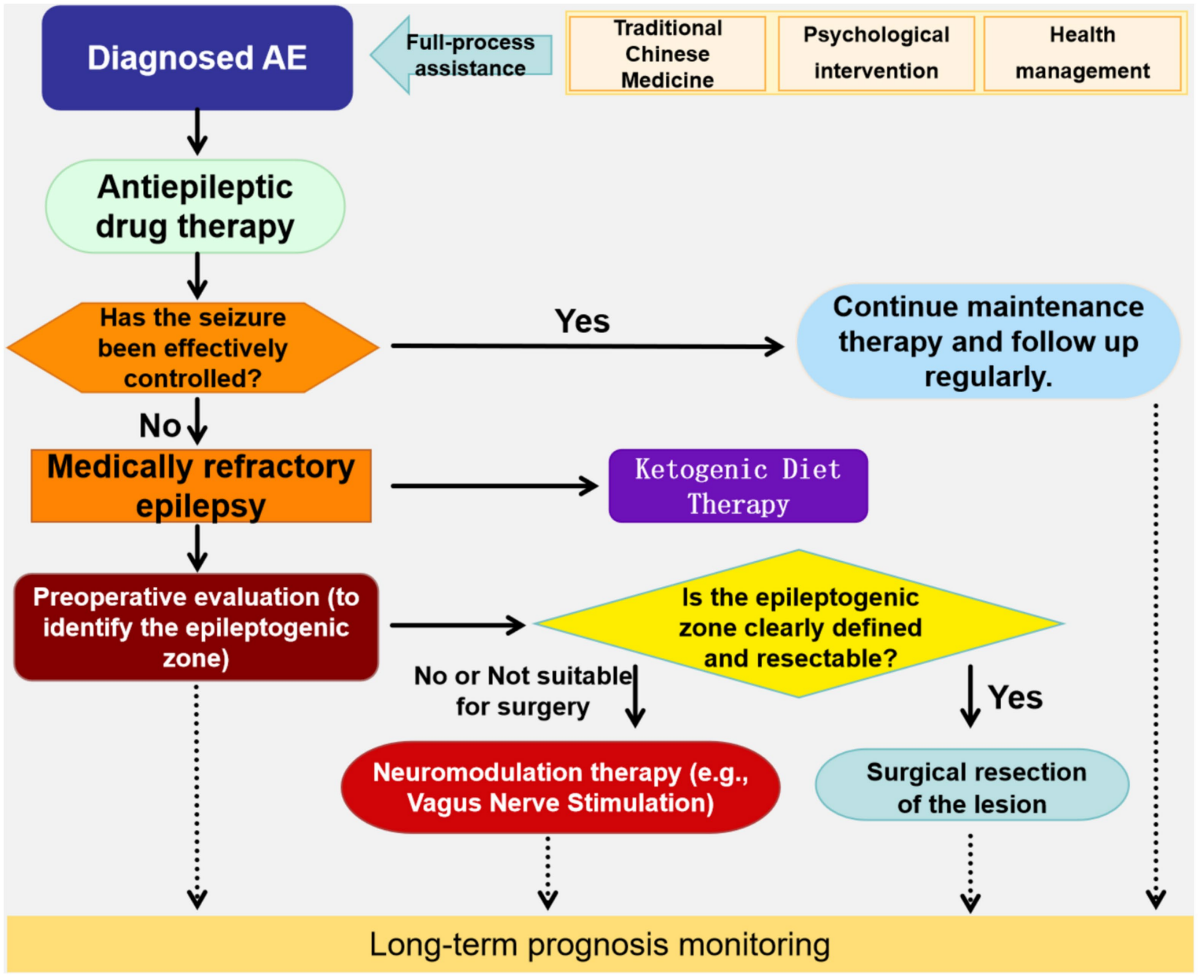
Flowchart for selecting treatment strategies for abdominal epilepsy in children (image created with PPT).

In addition to the aforementioned treatments, psychological intervention and health education are also important auxiliary measures. Due to the recurrent episodes and long duration of the disease, patients and their families may experience psychological issues, such as anxiety and depression (Xu et al., 2025). Psychological interventions, such as cognitive behavioral therapy (Ting, 2025b), can help alleviate psychological stress and improve treatment compliance. Health education should include knowledge about the disease, medication guidance, emergency measures for seizures, and daily care (Manzanares et al., 2024). It is recommended that patients keep a seizure diary, detailing the time of onset, duration, symptoms, and triggering factors, to help doctors adjust the treatment plan in a timely manner. In daily life, patients should avoid triggering factors, such as flashing stimuli, sleep deprivation, excessive fatigue, and emotional excitement, maintain regular sleep and a balanced diet, and limit caffeine intake (Amirhosseni et al., 2025). The process for selecting treatment strategies is shown in Figure 2.

## 9 Prognosis assessment and future perspectives

### 9.1 Prognosis

The commonly cited view of a generally favorable prognosis for pediatric AE is primarily derived from follow-up of published case series, which may be subject to significant publication bias (cases with good outcomes are more likely to be reported). While these reports suggest a tendency for spontaneous remission in adolescence for a proportion of children, the true natural history in an unselected population remains unclear. Long-term, prospective cohort studies are lacking. The prognosis for children with idiopathic epilepsy is better than for those with symptomatic epilepsy. Factors such as age of onset, specific epilepsy syndrome type, and etiology are closely associated with outcomes. A younger age at onset, symptomatic or cryptogenic epilepsy, and specific syndromes, such as infantile spasms, are often linked to a less favorable prognosis (Xiao, 2026). Several factors, such as the underlying cause, age at onset, seizure frequency and duration, seizure type, response to drug therapy, and the presence of complications, influence the prognosis of AE. Idiopathic epilepsy generally has a better prognosis compared to cases secondary to brain injury or genetic metabolic diseases. An early age of onset, high seizure frequency, and prolonged seizure duration are associated with a relatively poorer prognosis. Generalized tonic–clonic seizures and absence seizures typically have a better prognosis than complex partial seizures. A good response to anti-epileptic drugs is a positive prognostic indicator. Therefore, early diagnosis, standardized treatment, and regular follow-up are crucial for improving outcomes in pediatric AE. Follow-up should include periodic neurological examinations, EEG, and neuroimaging studies to assess neurological recovery and adjust treatment plans promptly. For patients with cognitive and behavioral abnormalities, early neuropsychological assessment and intervention are essential to improve function. It is also important to avoid potential triggers, such as sleep deprivation, flashing lights, and infections/fever, while avoiding overprotection or discrimination against the child.

### 9.2 Limitations and future perspectives

Despite the growing understanding of AE, several challenges remain to be addressed: ① The pathogenesis of AE requires further elucidation, particularly the specific neural circuits involved in autonomic manifestations. The roles of genetic factors and the gut–brain axis also warrant deeper investigation. It must be emphasized that most proposed mechanisms are supported by low-level evidence, such as animal models, small neuroimaging studies, and correlative findings. Further elucidation is needed, particularly the specific regulatory mechanisms. The roles of genetic factors and the gut–brain axis require more in-depth exploration using hypothesis-driven clinical and translational research designs. Furthermore, the potential role of advanced functional neuroimaging (PET and SPECT) in delineating the epileptogenic network and its consistency across AE patients remains largely unexplored due to the scarcity of dedicated studies. ② The lack of unified international diagnostic criteria for AE leads to inconsistencies in clinical diagnosis and affects the accuracy of epidemiological data. There is a need to establish international multicenter registries with standardized definitions and follow-up indicators. ③ Large-scale, multicenter clinical studies should be conducted to develop more precise diagnostic criteria and treatment guidelines. ④ Exploration of novel therapeutic targets is essential, such as neuromodulation techniques, gene therapy, and stem cell therapy, alongside the development of new anti-epileptic drugs. ⑤ Long-term follow-up studies are necessary to evaluate the sustained efficacy and safety of different treatment regimens, assess their long-term impact on neurodevelopment, and formulate personalized long-term management plans. ⑥ With the continuous advancement of artificial intelligence technology, its application in the diagnosis, seizure prediction, drug selection, and neuropsychological assessment of AE is expected to become increasingly precise (Yu S. T. et al., 2025). ⑦ Attention should be paid to the quality of life and social adaptation of affected children. Evidence-based management pathways involving shared decision-making among families, schools, and healthcare providers should be developed to provide comprehensive rehabilitation support. ⑧ The overall body of evidence on AE is of low quality. The literature is dominated by case reports, small retrospective case series, and expert opinion. There is a striking paucity of controlled studies, prospective cohorts, and randomized trials. This fundamental limitation underpins the uncertainties in epidemiology, diagnosis, and treatment discussed throughout this review and must be a primary focus for future research.

## 10 Conclusion

AE in children is a distinct type of epilepsy characterized primarily by paroxysmal abdominal pain. Its pathogenesis is closely associated with abnormal electrical discharges in the temporal and insular cortices. Diagnosis currently depends on a combination of suggestive clinical manifestations, ictal or interictal epileptiform abnormalities on EEG (ideally captured during an episode with video-EEG), and a positive response to antiepileptic drugs. Current treatment primarily involves anti-epileptic medication, with new advances also seen in approaches like the ketogenic diet and surgical intervention. The prognosis for most affected children is favorable. However, significant challenges remain, such as an incomplete understanding of its pathogenesis, the absence of validated diagnostic criteria, and a lack of high-quality evidence to guide treatment. Future efforts should focus on more extensive and in-depth research to further elucidate the mechanisms of AE, establish standardized diagnostic and treatment guidelines, and strengthen both basic and clinical studies. These steps are crucial for improving diagnostic and therapeutic outcomes and enhancing the long-term prognosis for children with AE.
